# Psychometric Characteristics and Sociodemographic Adaptation of the Corrigan Agitated Behavior Scale in Patients With Severe Mental Disorders

**DOI:** 10.3389/fpsyg.2021.779277

**Published:** 2021-12-08

**Authors:** María Elena Garrote-Cámara, Iván Santolalla-Arnedo, Regina Ruiz de Viñaspre-Hernández, Vicente Gea-Caballero, Teresa Sufrate-Sorzano, Pablo del Pozo-Herce, Rebeca Garrido-García, Esther Rubinat-Arnaldo, Raúl Juárez Vela

**Affiliations:** ^1^Mental Health Center of Albelda de Iregua, Riojan Health Service, Government of La Rioja, La Rioja, Spain; ^2^Group of Research in Sustainability of the Health System, Biomedical Research Center of La Rioja (CIBIR), Logroño, Spain; ^3^Care Research Group (GRUPAC) - University of La Rioja, La Rioja, Spain; ^4^Faculty of Health, Valencian International University, Valencia, Spain; ^5^Department of Psychiatry, Fundación Jiménez Díaz University Hospital, Madrid, Spain; ^6^Najera Health Center, Riojan Health Service, Government of La Rioja, La Rioja, Spain; ^7^Research Group of Health Care (GRECS) - IRBLleida, Department of Nursing and Physiotherapy, University of Lleida, Lleida, Spain; ^8^Research Group Society, Health, Education and Culture (GESEC), University of Lleida, Lleida, Spain; ^9^Center for Biomedical Research Network on Diabetes and Associated Metabolic Diseases (CIBERDEM), Carlos III Health Institute, Barcelona, Spain

**Keywords:** nursing, psychometrics, mental health, psychiatry, psychomotor agitation

## Abstract

**Background:** Severe mental disorder (SMD) is understood in a first approximation as a disorder of thought, emotion, or behavior of long duration, which entails a variable degree of disability and social dysfunction. One of the most widely used assessment scales for agitated behavior, in its English version, is the Corrigan Agitated Behavior Scale (ABS); several studies have demonstrated solid psychometric properties of the English version, with adequate internal consistency.

**Objective:** The objective of this study was to evaluate the psychometric properties of the Spanish version of the ABS Corrigan scale, in a sample of patients with severe mental disorders. The psychometric analyses of the Spanish version of the ABS Corrigan included tests of the reliability and validity of its internal structure.

**Results:** The structure of the factorial loads of the analyzed elements is consistent with the hypothesized three-dimensional construction referred to in the original ABS. The results suggest that the reliability and validity of the three dimensions are acceptable (First 0.8, Second 0.8, and Third 0.7). The internal consistency of the Spanish version of the complete ABS and of each of the three domains that compose it is high, with values very close to those found in the original version, with approximate figures of 0.9.

**Conclusion:** In our study, the three domains aim to explain 64.1% of the total variance of the scale, which exceeds the 50% found in the original version.

## Introduction

Severe mental disorder (SMD) is understood in a first approximation as a disorder of thought, emotion, or behavior of long duration, which entails a variable degree of disability and social dysfunction (Vila et al., 2007). In the Diagnostic and Statistical Manual of Mental Disorders (DSM-5), specifically, it is “a syndrome characterized by a clinically significant alteration of the cognitive state, emotional regulation or behavior of an individual, which reflects a dysfunction of psychological processes, biological or developmental that underlie their mental function” (American Psychiatric Association, 2013). According to the National Institute of Mental Health, SMD patients face a limitation in being able to carry out important activities in their daily lives. (National Institute of Mental Health, 2019). There are very diverse pathologies that can be included in the concept of serious mental disorder, and they are usually classified according to the symptoms, evolution, chronicity and level of disability they cause in the lives of patients (World Health Organization, 2018). Patients with SMD experience a significant deterioration, which limits the development of their daily activities (Hazelden Foundation, 2016; Padilla et al., 2018). At present, the two most used taxonomies for the diagnosis of SMD are the Classification of Mental and Behavioral Disorders (ICD-11) of the World Health Organization (2018) and the Diagnostic and Statistical Manual of Mental Disorders (DSM-5) of the American Psychiatric Association (2013). The main groups of diagnoses that give rise to the appearance of situations valued as a severe mental disorder are as follows: schizophrenia and a group of psychotic disorders; depressive disorder, manic disorder and bipolar disorder; anxiety disorders; and personality disorders (Pichot et al., 1995; Gobierno de Navarra, 2005). In Spain, the results of the 2017 National Health Survey (Ministry of Health, Consumer Affaris and Social Welfare, 2017) indicate that 10.8% of adults reported having been diagnosed with a mental health problem; women report a mental health problem more frequently than men, 14.1% versus 7.2%. Furthermore, 6.7% of adults report chronic anxiety, 9.1% of women and 4.3% of men. Depression is declared in the same proportion as anxiety (6.7%), and it is more than double in women (9.2%) than in men (4%).

All the diagnoses described above present a series of common characteristics, such as greater vulnerability to stress, difficulties in coping with the demands of the environment, problems in managing frustration, deficits in their abilities to manage autonomously, difficulties in social interaction and high dependence on other people and health and/or social services (Vila et al., 2007; Hazelden Foundation, 2016; Padilla et al., 2018). Symptoms of the disease and difficulties in interacting with the environment develop complications, such as psychomotor agitation, more prevalent in people suffering from a serious mental disorder. The prevalence of psychomotor agitation is high, up to 10% of patients seen in general emergency services (Pacciardi et al., 2013) and between 20 and 50% if we specifically consider psychiatric emergencies (Allen and Currier, 2004; Marco and Vaughan, 2005). Different studies relate psychomotor agitation with the suffering of a severe mental disorder (Bogner et al., 1999; Huber et al., 2016; Ostinelli et al., 2019), diverse psychiatric conditions, as well as some medical disorders (Galián Muñoz et al., 2011). Agitation of psychiatric origin is seen more frequently in patients with psychotic disorders, such as schizophrenia, schizoaffective disorder and the manic phase of bipolar disorder, as well as in some personality disorders (Feldman et al., 2001; Buckley et al., 2003; Battaglia, 2005; Serretti and Olgiati, 2005; Wittchen et al., 2011). In a manic episode, psychomotor agitation comes from the same phenomenology of the condition, where it is described as part of the symptom complex of mania with excessive motor and cognitive activity (Soutullo et al., 2011). In Spain, it is estimated that 25% of patients with schizophrenia and 15% of patients with bipolar disorder could suffer from at least one episode of agitation each year, with an average of two episodes per patient (Viñado et al., 2015).

Psychomotor agitation is characterized as a non-specific syndrome, of multifactorial etiology, which entails an alteration of motor behavior, consisting of a disproportionate and disorganized increase in motor functions and which may be accompanied by vegetative activation (profuse sweating, tachycardia, and mydriasis), severe anxiety, aggressiveness, panic, behavior lability, disinhibition or other intense emotional states (Allen and Currier, 2004; Pacciardi et al., 2013). The patient’s state of mind can manifest itself as nervous, euphoric and choleric, and laughter, crying and uncontrollable screaming are frequent, which can lead to verbal and/or physical aggressions, and therefore, pose a serious risk to both the patient themself (self-harm), as well as family members, healthcare personnel and the environment in general (hetero-harm) (Marco and Vaughan, 2005). On many occasions, the management of agitation presents difficulties, since they are usually people who are difficult to collaborate and who can present aggressive behaviors, disinhibition, which greatly hinders the assistance work, treatment and care of the patient.

Psychomotor agitation corresponds to one of the most important hospital emergencies in the care of severe mental disorder since it requires immediate professional assistance, and the safety of the patient is seriously compromised (Bogner et al., 2001; Buckley et al., 2003; Nott et al., 2006; Galián Muñoz et al., 2011; Ostinelli et al., 2019).

A large proportion of older people with mental disorders live at home and are cared for by their relatives, which requires the involvement of primary care physicians and rapid intervention (Adriaenssens et al., 2019). Whenever possible and when required by the patient, the patient should be assessed and cared for in their own environment, limiting trips to health centers for emergencies or seriousness, or for special tests (Snowdon, 2007). Home assessment should involve primary care professionals and should always take into account socio-demographic, somatic, functional and social aspects (Olivera et al., 2011). The assessment should result in the formulation of a care plan with clear objectives and defined responsibilities for members of the multidisciplinary team and the primary care team (Hoedemakers et al., 2019).

The evaluation of psychomotor agitation, as well as its management, constitutes a challenge for health professionals since this syndrome usually overwhelms their management skills. One of the most widely used assessment scales for agitated behavior, in its English version, is the Corrigan Agitated Behavior Scale (ABS; Corrigan, 1989). This scale provides both quantitative and qualitative data, determining the level of agitation and its characteristics at the level of lability, disinhibition and aggressiveness associated with the agitation episode (Corrigan, 1989; Corrigan and Bogner, 1994). It was designed by Corrigan (1989) to evaluate agitation in the context of patients in a period of post-encephalic trauma; however, since its design, it has been widely used in the evaluation of agitation in patients with severe mental disorders (Bogner et al., 1999; García et al., 2002; García - Ribera, 2014).

Given the importance of psychomotor agitation among the population suffering from a severe mental disorder and Spanish being the second most spoken language in the world (Instituto Cervantes, 2020), it is necessary to have a tool with which to assess agitation in these patients. The objective of this study was to evaluate the psychometric properties of the Spanish version of the ABS Corrigan scale in a sample of patients with severe mental disorder.

## Materials and Methods

### Translation, Adaptation and Modeling

Before testing its psychometric properties, the Corrigan ABS scale was translated and culturally adapted from its original English version into Spanish. We follow the guidelines published by Beaton et al. (2000) who divided the process into the following six steps: (1) translation, (2) synthesis, (3) back-translation, (4) back-translation synthesis, (5) review by the expert committee of the translated version, and (6) preliminary tests.

The original ABS Corrigan scale was translated into Spanish by two independent translators, an expert in medical translation and a researcher familiar with the instrument and its characteristics (forward translation). The translators were instructed to use simple sentences and avoid metaphors, colloquial terminology, passive sentences, and hypothetical statements. In a meeting of the expert committee, made up of the authors and the translators, the differences between the two translations were discussed, and the initial translation was unified (reconciliation). This first Spanish translation was blindly re-translated into English (back-translation). Subsequently, the expert committee compared and contrasted the original and retro-translated versions, in order to achieve the most precise adaptation possible to the original language of the Spanish version and agreed, by consensus, on the final Spanish version of the ABS Corrigan to be used in the sample.

Finally, as it is a hetero-applied administration instrument, cognitive interviews were completed in a sample of 20 nursing professionals (cognitive debriefing) that confirmed the legibility of the elements as they were written.

### Procedures

This study was conducted in the north region of Spain (La Rioja) using a cross-sectional design. We enrolled a sample of *n* = 140 participants admitted to the Mental Health of Albelda de Iregua (La Rioja) who met the following inclusion criteria: (1) being diagnosed severe mental disorder, (2) be admitted to psychiatric hospitalization units at the Rioja Health Service, (3) present an episode of psychomotor agitation between 2015 and 2020, and (4) being 18 years or older. According to Norman and Streiner (1996) we enrolled 140 participants at least five patients for item of the scale. All data were collected by qualified nurses, who had been specifically trained for this purpose, during the patients’ admission.

The sample size was estimated according to the criteria to perform a factor analysis that contemplated a minimum of 10 subjects for each item (De Vet et al., 2005). The Corrigan Agitated Behavior Scale is a hetero-applied administration instrument, where the interviewer must evaluate 14 items, which are quantified with a Likert-type scale according to the observation of the professional (Bogner et al., 1999; García et al., 2002; García - Ribera, 2014). The scale was completed by trained nursing professionals; the psychomotor agitation data of patients with severe mental disorders, who developed an episode of psychomotor agitation during their admission to the psychiatric hospitalization units of the Rioja Health Service, were collected. The inclusion criteria were patients diagnosed with a severe mental disorder, of both sexes, older than 16 years, admitted to the psychiatric hospitalization units of the Rioja Health Service, and who developed an episode of psychomotor agitation between 2015 and 2020.

The scale includes 14 items that are grouped in the original English version into three factors: disinhibition, items 1, 2, 6, 7, 8, 9, and 10; aggressiveness, items 3, 4, 5, and 14; lability, items 11, 12, and 13 (Corrigan, 1989). There are 14 items that assess the ability to sustain attention: impulsiveness, pain tolerance and frustration management; cooperation and demand; violence and threats; explosiveness, anger and unpredictability; self-stimulating behaviors; pull objects or ties from the bed; roams the treatment areas; restlessness and excessive movement; repetitive behaviors; excessive, fast and loud language; sudden mood change; excessive facility for crying and/or laughing; self-injurious behaviors. The professional must score according to a Likert-type scale of 4 degrees of intensity, from 1 (absence) to 4 (extreme degree). The sum of the scores of the 14 items determines the severity of the agitation; the higher the score, the greater the severity. The score in each of the three factors determines if the episode is characterized more by its lability, disinhibition, or aggressiveness (Corrigan, 1989; Teri et al., 1992; Corrigan and Bogner, 1994; Spanish Society of Emergency and Emergency Nursing, 2009). In addition to the score for each item, socio-demographic data and the main diagnosis that led to admission were collected.

### Statistical Analysis

Sociodemographic and clinical variables were analyzed using descriptive statistics as the mean and standard deviation in the case of quantitative variables and frequencies in the case of categorical variables. In addition, other parametric techniques, including the mean, standard deviation, skewness, and kurtosis, were used to describe responses to items and summarize the total scale score. The psychometric analyses of the Spanish version of the ABS Corrigan included tests of reliability and validity of its internal structure. We evaluated the reliability of the scale by exploring internal consistency, and we calculated Cronbach’s alpha coefficient of the global scale and each of the theoretical dimensions, accepting significance values of 0.70 or higher as an indicator of good internal consistency (Argimon, 2012).

We evaluated the underlying factor structure of the ABS scale in Spanish using factor analysis. To assess the relevance of performing an exploratory factor analysis on the sample, the Kaiser–Meyer–Olkin (KMO) sample adequacy statistic and the Bartlett sphericity statistic were previously calculated. The suitability of the analysis was determined with a KMO greater than 0.6, and in the case of Bartlett’s test of sphericity, a rejection of the null hypothesis of sphericity of *p* < 0.05 to ensure that the use of the factorial model was adequate. An exploratory factor analysis (EFA) was performed using principal component analysis with a Varimax rotation to determine the number of latent constructs and the underlying factorial structure of domains on the ABS scale in its Spanish version. The number of factors on the scale was estimated, considering two complementary criteria: (1) the Kaiser–Guttman or latent root criterion and (2) the drop contrast criterion (Hair et al., 2005; Martínez et al., 2006; Tabachnick et al., 2007). The statistical software used was SPSS (IBM SPSS Statistics for Windows, Version 23.0, IBM Corp., Armonk, NY, United States).

### Ethical Considerations

The data collection was anonymous and did not collect personal data or devices that could identify the informant. The information was treated confidentially and anonymously since they had dissociated data, following the Data Protection Regulation (EU) 2016/679 of the European Parliament and the Spanish Organic Law 3/2018. The study was approved by the Ethics Committee of the Rioja Biomedical Research Center (CIBIR) (reference CEImLar P.I. 467) (Biomedical Research Center of La Rioja, 2021). The researchers do not declare any type of ethical, moral or legal conflict, nor do they claim to have received financial compensation of any other kind. The participants did not receive any type of compensation for answering the questionnaire, as it was voluntary.

## Results

Table 1 illustrates the main sociodemographic characteristics of the sample. The mean age of the sample was 45.61 years. In total, 52.9% of the sample were men, and 47.1% were women. Regarding the underlying pathology, 60.7% of the sample presented schizophrenia and other psychotic disorders, of which 60% were men and 40% were women; 9.3% presented a depressive episode, of which 30.8% were men and 69.2% were women; 2.1% presented a manic episode, all of which were women; 8.6% presented a bipolar episode, of which 33.3% were men and 66.7% were women; 10.7% presented a personality disorder, of which 40% were men and 60% were women; 8.6% presented other pathologies, of which 75% were men and 25% were women.

**TABLE 1:** Main sociodemographic characteristics of the sample (*n* = 140).

Variables	*n*	%
**Age**		
<18	1	0.7
18–30	21	15
31–50	69	49.3
51–65	28	20
66–79	17	12.1
>80	4	2.9
**Sex**		
Women	66	47.1
Men	74	52.9
**Mental illness**		
Schizophrenia and other psychotic disorders	85	60.7
Depressive episode	13	9.3
Manic episode	3	2.1
Bipolar episode	12	8.6
Personality disorder	15	10.7
Others	12	8.6

The mean, standard deviation (SD), asymmetry and kurtosis values for each of the items of the Spanish version of the scale of psychomotor agitation, ABS Corrigan, are indicated in Table 2. Most of the items are distributed normally, without excessive asymmetry and kurtosis. The items with the highest scores are item 2 “Impulsive, impatient, tolerates pain or frustration poorly” and item 3 “Uncooperative, does not let them take care of him, demanding.” The lowest scores were recorded by item 7 “Pulls the tubes or ties on the bed” and item 13 “Cries or laughs easily and excessively.”

**TABLE 2:** Descriptive statistics of the items of the Spanish version of the ABS Corrigan scale.

Items	Mean	SD±	Asymmetry	Kurtosis
1. Poor attention paid, easily distracted, inability to concentrate	2.8	± 0.8	−0.3	−0.3
2. Impulsiveness, impatience, poor tolerance for pain and frustration	3.2	± 0.8	−1	0.4
3. Little cooperation, does not allow to be taken care of, demanding	3.1	± 0.8	−0.6	−0.2
4. It is violent, threatens people and property	2.9	± 1	−0.5	−0.8
5. Explosive or unpredictable outbursts of anger	2.9	± 0.9	−0.6	−0.6
6. Rocks, rubs, groans, or exhibits other self-stimulating behavior	2.2	± 1.1	0.3	−1.2
7. Pulls objects or ties from the bed	1.7	± 1	1	−0.4
8. Roams through treatment areas	2.5	± 1.1	−0.1	−1.3
9. Restlessness, it comes and goes, it moves excessively	2.8	± 0.9	−0.5	−0.5
10. Repetitive, motor, or verbal behaviors	2.7	± 0.9	−0.3	−0.8
11. Speak fast, loud, or excessively	2.9	± 1	−0.5	−0.9
12. Sudden mood changes	2.8	± 1	−0.6	−0.7
13. Cries or laughs easily and excessively	2	± 1	0.6	−0.8
14. It hurts or insults	2.7	± 1	−0.3	−1.1

In terms of reliability, the internal consistency of the Spanish version of the ABS Corrigan scale and its domains is excellent (Alexandre et al., 2013; Coluci et al., 2015; Cunha et al., 2016). Cronbach’s alpha for the total scale is 0.9, and for each of the scale dimensions, disinhibition is 0.8, aggressiveness is 0.8 and lability is 0.7 (see Table 3).

**TABLE 3:** Cronbach’s Alpha – ABS Corrigan. Scale dimensions.

Dimension	Cronbach’s Alpha
Disinhibition	0.8
Aggressiveness	0.8
Lability	0.7
Total	**0.9**

*Cronbach’s alpha for the total scale is 0.9.*

The adequacy tests, before performing the exploratory factor analysis, resulted in a Kaiser–Meyer–Olkin (KMO) value of 0.9. Bartlett’s test of sphericity is significant (*p* < 0.01) both in the analysis of each of the domains and in the global analysis of the scale. Sampling suitability is high in the exploratory factor analysis.

**FIGURE 1:**
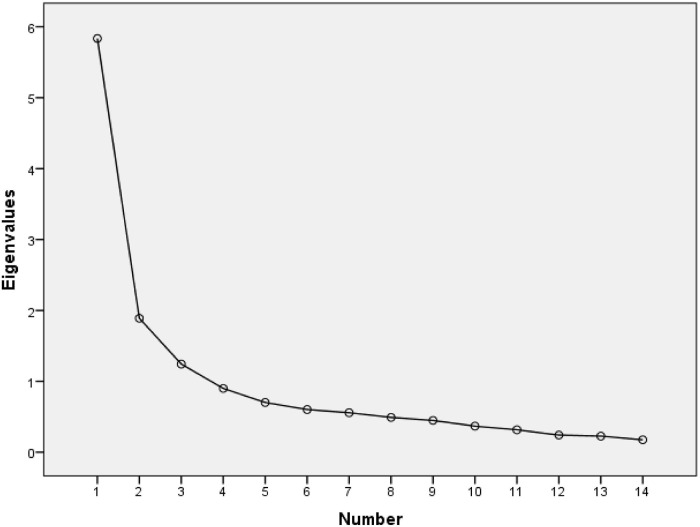
Sedimentation chart of the Spanish version of the ABS Corrigan.

The Kaiser–Guttman or latent root criterion identified three factors with eigenvalues greater than 1, as shown in Table 4, which would explain 64.1% of the total variance of the items. The second criterion, fall contrast or screen test, also showed through the sedimentation graph the presence of three factors, as reflected in Figure 1.

**TABLE 4:** Total explained variance – ABS Corrigan. Sum of rotation of charges square.

Component	Total	% Variance	% Accumulated
1	3.9	27.9	27.9
2	3.5	25.3	53.2
3	1.5	10.9	64.1

The exploratory factor analysis (EFA) was developed using principal component analysis with a Varimax rotation, considering the following criteria: factor load >0.30, the number of items per factor according to the original version, the interpretability of the results and the theory that supports the ABS Corrigan scale. According to these criteria, the best solution was to identify three dimensions of grouping the items as in the original version; see Table 5. The dimension “disinhibition” included items 1, 2, 6, 7, 8, 9, and 10; the “aggressiveness” dimension included items 3, 4, 5, and 14; the dimension “lability” items 11, 12, and 13. In the matrix of the rotated component (Varimax) of Table 5, it is observed that the elements load significantly in the three previous factors.

**TABLE 5:** Rotated component matrix. Principal component analysis (Varimax).

Item	Factor 1	Factor 2	Factor 3
Item 1	0.8		
Item 2	0.5		
Item 3		0.4	
Item 4		0.9	
Item 5		0.9	
Item 6	0.7		
Item 7	0.6		
Item 8	0.5		
Item 9	0.7		
Item 10	0.8		
Item 11			0.3
Item 12			0.4
Item 13			0.8
Item 14		0.7	

The results of the exploratory factor analysis (EFA) suggest that the three dimensions determined in the ABS Corrigan evaluation scale originally validated in the US, “disinhibition,” “aggressiveness,” and “lability,” are applicable for this version in Spanish. The structure of the factorial loads of the analyzed elements is consistent with the hypothesized three-dimensional construction referred to in the original ABS. The results suggest that the reliability and validity of the three dimensions is acceptable. The results are largely consistent with the initial hypothesis.

## Discussion

The adaptation and cross-cultural validation into Spanish of the Agitated Behavior Scale (ABS Corrigan) was carried out, thus obtaining a culturally equivalent instrument that allows the evaluation of psychomotor agitation in patients with severe mental disorder, obtaining levels of severity of agitation, such as the exploration of three related domains, disinhibition, lability, and aggressiveness. The results of this study indicate that these three domains are applicable in patients with severe mental disorder, and the reliability and validity measures are satisfactory for their use in Spanish.

During the cross-cultural adaptation process, there were no language difficulties; only some expressions were slightly modified to ensure their cultural equivalence. The nursing professionals who participated in the study did not show any difficulties in filling in the scale after attending to and observing the patient’s psychomotor agitation. As it is a hetero-applied administration instrument, the patients do not need to understand the scale.

Several studies have demonstrated the solid psychometric properties of the English version, with an adequate internal consistency, Cronbach’s alpha between 0.801 and 0.921 (Corrigan, 1989; Corrigan and Bogner, 1994; Bogner et al., 1999; Vilibiæ et al., 2014; Hellweg and Schuster-Amft, 2016). The internal consistency of the Spanish version of the complete Corrigan Agitated Behavior Scale and of each of the three domains that compose it is high, with values very close to those found in the original version, with approximate figures of 0.9. Similar to the original English version, and the German version of the scale, Cronbach’s alpha of the aggression, disinhibition and lability subscales is lower than that of the total scale score, indicating that the total score remains the best overall measure of agitation (Corrigan, 1989; Corrigan and Bogner, 1994; Hellweg and Schuster-Amft, 2016). In our study, the three adopted domains explain 64.1% of the total variance of the scale, which exceeds the 50% found in the original version (Corrigan, 1989).

The exploratory factor analysis of this study supports the maintenance of the same three domains adopted in the original version and confirmed in the validated version in German (Corrigan, 1989; Corrigan and Bogner, 1994; Hellweg and Schuster-Amft, 2016). Although adequate correlations were observed in the German version, the correlations were lower than those in the original English version and in this study, in which we analyzed a Spanish version. This may be due to the fact that the German study was multidisciplinary compared to the Spanish version, which is monodisciplinary. The nursing professional knows the patient, is responsible for the patient’s health care, knows the signs and symptoms of the disease and is the first line of care for episodes of psychomotor agitation. The fact that these professionals assess the agitation episodes through the 14 items of the ABS Corrigan improve the adequacy of the results of the scale (Santolalla et al., 2015; Caqueo-Urízar et al., 2016; Hellweg and Schuster-Amft, 2016).

In the exploratory factor analysis, it is observed, for example, that there are items related to the aggressiveness dimension, such as item 4 “is violent, threatens person or property,” item 5 “explosive or with unpredictable anger attacks” and item 14 “harm or insult,” which highly identified with the dimension, as also observed in the study of the German version (Hellweg and Schuster-Amft, 2016). The aggressiveness dimension is perhaps one of the most related to psychomotor agitation in patients with severe mental disorder, especially regarding the diagnostic group of schizophrenia and other psychotic disorders, which helps healthcare professionals to evaluate the items related to this dimension (Lehman et al., 2004). In fact, psychomotor agitation is particularly prevalent among the population with schizophrenia and bipolar disorder (Witt et al., 2013; Zhou et al., 2015; Vieta and Garriga, 2016; Wu et al., 2018). In Spain, a recent report indicated that 25% of patients with schizophrenia and 15% of those with bipolar disorder could be expected to experience at least one episode of psychomotor agitation each year (Vieta et al., 2017). On the other hand, the lower load of item 3 “Uncooperative, does not let them take care of him, demanding” on the aggressiveness dimension can be related to cultural and translation factors, as the demand or little collaboration is not significantly related in our country with aggressiveness, so the nursing professional could score this item in a more random way (Real Academia Española, 2021).

In this line, item 8 “wanders through the treatment areas,” item 11 “speaks fast, loud, or excessively,” and item 12 “changes of mood suddenly,” with lower loads on its dimensions, can be more difficult to identify with its domain due to cultural factors or translation difficulties. Describing these items further could improve the response from professionals. These notes do not substantially change the use of the scale in its Spanish version, but they do inform us about the importance of not only the linguistic but also the cultural validation of the tools for measuring concepts as complex as agitation.

Psychomotor agitation is characterized by an interruption in the relationship or collaboration of the health professional and the patient, severely interfering with the evaluation, treatment, health care and prognosis of the underlying pathology. The management and care of the agitated patient constitute a first level health care demand. The safety of the patient, health care personnel and third parties; reducing the progression of the condition by acting on prodromal phases; and limiting complications during crises by their early discovery and management, must be guaranteed. The availability of a psychomotor agitation assessment scale, adapted to the language and culture of our country, with adequate psychometric properties, offers greater possibilities for prevention, adequate diagnosis, research, treatment and effective care, improving the prognosis and quality of life of affected patients (Santolalla et al., 2015; Caqueo-Urízar et al., 2016).

## Limitations

The sample used for data validation is sufficiently large to guarantee an adequate representation of patients with severe mental disorder. However, most of the sample for this study presents schizophrenia, in this sense it is recommended to focus the analysis only on these patients, since the rest of the sample is too stratified in other pathologies.

## Conclusion

The results of this translation and validation study into the Spanish version of the Agitated Behavior Scale (ABS Corrigan) suggest that the reliability and validity of the three dimensions is acceptable, with data similar to the original English version and better fit than the German version (Corrigan, 1989; Hellweg and Schuster-Amft, 2016). The total scale score remains the best overall measure of agitation. The results are largely compatible with the initial hypothesis, which makes them useful for clinical and research use in our country.

## Relevance for Clinical Practice

The use of validated instruments means providing health professionals with reliable and valid tools. Different studies have demonstrated the solid properties of the Corrigan Agitated Behavior Scale in its English version. However, no studies have validated this scale in the Spanish language. With this study, we reveal psychometric characteristics and sociodemographic adaptation of the Corrigan Agitated Behavior Scale in patients with various mental disorders to one of the most widely spoken language in the world.

## Data Availability Statement

The original contributions presented in the study are included in the article/supplementary material, further inquiries can be directed to the corresponding author.

## Ethics Statement

The study was approved by the Ethics Committee of the Rioja Biomedical Research Center (CIBIR) (reference CEImLar P.I. 467) (Centro de Investigación Biomédica, 2021). Written informed consent from the patients/participants was not required to participate in this study in accordance with the national legislation and the institutional requirements.

## Author Contributions

All authors listed have made a substantial, direct, and intellectual contribution to the work, and approved it for publication.

## Conflict of Interest

The authors declare that the research was conducted in the absence of any commercial or financial relationships that could be construed as a potential conflict of interest.

## Publisher’s Note

All claims expressed in this article are solely those of the authors and do not necessarily represent those of their affiliated organizations, or those of the publisher, the editors and the reviewers. Any product that may be evaluated in this article, or claim that may be made by its manufacturer, is not guaranteed or endorsed by the publisher.
